# Pulmonary Embolism Response Teams—Evidence of Benefits? A Systematic Review and Meta-Analysis

**DOI:** 10.3390/jcm13247623

**Published:** 2024-12-14

**Authors:** Amelia Bryan, Quincy K. Tran, Jalil Ahari, Erin Mclaughlin, Kirsten Boone, Ali Pourmand

**Affiliations:** 1 Department of Emergency Medicine, School of Medicine and Health Sciences, The George Washington University, Washington, DC 20037, USA; aybryan@gwu.edu (A.B.); erin.mclaughlin@gwmail.gwu.edu (E.M.); kboone@mfa.gwu.edu (K.B.); 2 Department of Emergency Medicine, School of Medicine, University of Maryland, Baltimore, MD 21201, USA; 3 Program in Trauma, The R Adam Cowley Shock Trauma Center, School of Medicine, University of Maryland, Baltimore, MD 21201, USA; 4 Pulmonary and Critical Care Medicine, School of Medicine and Health Sciences, The George Washington University, Washington, DC 20037, USA; jahari@mfa.gwu.edu

**Keywords:** pulmonary embolism, PERT, catheter-directed therapy, thrombolysis PE

## Abstract

**Background:** Venous thromboembolisms constitute a major cause of morbidity and mortality with 60,000 to 100,000 deaths attributed to pulmonary embolism in the US annually. Both clinical presentations and treatment strategies can vary greatly, and the selection of an appropriate therapeutic strategy is often provider specific. A pulmonary embolism response team (PERT) offers a multidisciplinary approach to clinical decision making and the management of high-risk pulmonary emboli. There is insufficient data on the effect of PERT programs on clinical outcomes. **Methods:** We searched PubMed, Scopus, Web of Science, and Cochrane to identify PERT studies through March 2024. The primary outcome was all-cause mortality, and the secondary outcomes included the rates of surgical thrombectomy, catheter directed thrombolysis, hospital length of stay (HLOS), and ICU length of stay (ICULOS). We used the Newcastle−Ottawa Scale tool to assess studies’ quality. We used random-effects models to compare outcomes between the pooled populations and moderator analysis to identify sources of heterogeneity and perform subgroup analysis. **Results:** We included 13 observational studies, which comprised a total of 12,586 patients, 7512 (60%) patients were from the pre-PERT period and 5065 (40%) patients were from the PERT period. Twelve studies reported the rate of all-cause mortality for their patient population. Patients in the PERT period were associated with similar odds of all-cause mortality as patients in the pre-PERT period (OR: 1.52; 95% CI: 0.80–2.89; *p* = 0.20). In the random-effects meta-analysis, there was no significant difference in ICULOS between PERT and pre-PERT patients (difference in means: 0.08; 95% CI: −0.32 to 0.49; *p* = 0.68). There was no statistically significant difference in HLOS between the two groups (difference in means: −0.82; 95% CI: −2.86 to 1.23; *p* = 0.43). **Conclusions:** This meta-analysis demonstrates no significant difference in all studied measures in the pre- and post-PERT time periods, which notably included patient mortality and length of stay. Further study into the details of the PERT system at institutions reporting mortality benefits may reveal practice differences that explain the outcome discrepancy and could help optimize PERT implementation at other institutions.

## 1. Introduction

Venous thromboembolisms including pulmonary embolism (PE) and deep-vein thrombosis (DVT) represent a major cause of morbidity and mortality in the US, affecting as many as 900,000 people in the US each year and accounting for up to 100,000 deaths annually [1]. In the US, the incidence of PE is between 60 and 120 per 100,000 people; annually, 60,000 to 100,000 deaths are attributed to PE [2]. There is a broad range of presentations of PE from those that are incidentally found to those that cause hemodynamic instability and cardiac arrest. Reflective of this diverse set of presentations, there are also a variety of treatment options spanning from outpatient management with oral anticoagulants to more invasive inpatient methods such as catheter-directed thrombolysis, surgical interventions, or extracorporeal membrane oxygenation (ECMO) [3,4].

When selecting appropriate treatment methods for pulmonary emboli, there can be a degree of inter-provider variability. At the sub-massive PE level, most societal guidelines regarding intervention selection, specifically regarding whether or not to use thrombolytics, require weighing a variety of clinical indicators such as evidence of right ventricular dysfunction, biomarker elevation, respiratory insufficiency, and patient-specific comorbidities [5,6,7]. In massive PEs, there is a greater degree of consensus from society’s recommendations, with thrombolytics generally recommended and catheter-directed therapies being reserved for cases where thrombolytics have failed or are contraindicated [5,6,7]. However, controversy still exist, whereas invasive therapies that are considered first-line therapy, are still left at the discretion of the treating physician [6].

A relatively novel and emerging way to treat diagnosed massive or sub-massive PE involves a pulmonary embolism response team (PERT), which is a group of healthcare professionals from multiple disciplines that quickly respond to high-risk PEs by assessing the patient’s risk and initiating treatments [8]. The PERT model was originally developed at Massachusetts General Hospital in 2012 and was designed based on other rapid-response teams, such as teams for in-hospital cardiac arrests and ST-segment elevation myocardial infarctions [9]. The need for PERTs is multifold due to the complexity and differences in each PE, the importance of rapid intervention due to hemodynamic and cardiopulmonary instability, and the extensive treatment modalities that span multiple medical disciplines [9]. PERTs unite specialists from a variety of fields to evaluate patients with high-risk PEs on a case-by-case basis to determine the best treatment modality. While team composition varies between institutions, PERTs are usually composed of representatives from emergency medicine, critical care, interventional radiology, interventional cardiology, cardiothoracic surgery, or vascular surgery [4,10].

Many institutions who have established a PERT process have begun to analyze the effect of the PERT approach on treating patients with PEs at a single center level. However, there are minimal data and thus a paucity of research investigating the outcomes of PERT as whole [11]. This systematic review and meta-analysis aims to investigate if PERTs improves the all-cause mortality for patients with pulmonary embolism.

## 2. Methods

### 2.1. Study Selection

This systematic review and meta-analysis was conducted in accordance with the Preferred Reporting Items for Systematic Review and Meta-Analysis (PRISMA) protocols [12]. The Patient, Intervention, Comparison, Outcome (PICO) format framework was used.

Patient: Patients with any findings of pulmonary embolism.

Intervention: Patients who were treated under the guidance of a PERT (post-PERT).

Control: Patients who were not treated under the guidance of a PERT (pre-PERT).

Outcome: The primary outcome was all-cause mortality at the earliest time frame being used by the authors. This outcome was selected because it was the most commonly reported outcome in the PERT literature. Secondary outcomes included the rates of surgical thrombectomy, catheter-directed thrombolysis, hospital length of stay (HLOS), and ICU length of stay (ICULOS). We did not consider endovascular or catheter-directed thrombectomies as a secondary outcome in our analysis as they are relatively novel. We anticipated a small number of studies. Additionally, since they are novel, these interventions would be mostly bias toward more recent post-PERT periods, while these techniques were not widely available for historical patients in the pre-PERT period.

The PERT timelines were categorized as defined by the respective studies. Specifically, the “pre-PERT” period refers to the timeframe before the implementation of the PERT, during which traditional management practices were employed. Conversely, the “post-PERT” period encompasses the timeframe following the establishment and activation of the PERT, reflecting the impact of this specialized multidisciplinary approach. Randomized control trials, quasi-experimental studies (pre- and post- intervention), and all observational studies (both prospective or retrospective studies) were eligible. The PERT structure is controlled by individual institutions, and there was no inclusion or exclusion criteria based on PERT provider composition. Adult patients with ages ≥ 18 years were eligible. Studies that were not published in English were excluded, as well as those without full text (conference proceedings, abstracts) and any non-original studies (review, meta-analysis).

### 2.2. Search Criteria

The topic was searched in PubMed, Scopus, Web of Science, and Cochrane from inception to 31 March 2024. The search term was available in Appendix A. The search term was reported as below. This study was registered with PROSPERO (CRD42023457239).

### 2.3. Study Selection and Data Extraction

Two investigators independently reviewed the titles and abstracts according to the pre-defined inclusion and exclusion criteria. Abstracts required agreement from both investigators to move forward to the next stage. When a disagreement occurred, both investigators would discuss among themselves to resolve it before involving a third investigator. The same process was utilized for the full-text review for inclusion in the final analysis. All screenings were conducted through Covidence (www.covidence.org, Melbourne, Australia).

Data were extracted to a standardized Excel spreadsheet (Microsoft Corp., Redmond, WA, USA). The diagnosis of PE was based on imaging modalities in seven studies, while the remaining studies relied on ICD-9 and ICD-10 codes as well as billing claims. Two investigators independently extracted studies’ information such as study design, sample size, types of interventions (surgical thrombectomy, catheter-directed thrombolysis [CDT]), and outcome (mortality, HLOS, and ICULOS). Data for other interventions such as thrombolytics, inferior vena cava filter, any anticoagulation, and any extra corporeal membrane oxygenation were also collected. We did not contact any authors for more data. The extracted data were reported as a consensus between investigators, so we did not calculate inter-rater agreement.

### 2.4. Quality Assessment

The Newcastle–Ottawa scale (NOS) was used to assess the quality of observational studies [13]. For each observational study, the NOS awards up to 9 points for each observation study; a score ≥ 7, for high-quality studies; and moderate- and low-quality studies have scores of 4–6 and ≤3, respectively. The overall risk of bias from the NOS was predominantly low.

Each individual study was graded according to the domain with the most risk of bias. Studies were assessed independently by two investigators. Any disagreement was resolved by consensus between the investigators and by a third investigator if needed.

### 2.5. Statistical Analysis

Random-effect meta-analyses were performed for all outcomes of interests. Outcomes were eligible for meta-analysis if they were reported in three or more studies. We used descriptive data with mean (±Standard Deviation [SD]) percentages to describe our data. Continuous data that were reported as the median and interquartile range [IQR] were converted to the mean and SD using a converter [14]. We reported the categorical outcome between pre-PERT and post-PERT as odds ratios and 95% confidence intervals. For the continuous outcomes, we expressed the results as mean difference and 95% confidence intervals. The primary outcome of all-cause mortality was evaluated using sensitivity analysis with random-effects meta-analysis with one study removed. In this sensitivity analysis, each individual study was sequentially omitted from the random-effects meta-analysis. Thus, the sensitivity analysis demonstrated any outlying study or individual studies that single-handedly affected the overall effect size. Random-effects meta-analysis with one study removed was performed for sensitivity analysis. Sensitivity analyses were performed for two outcomes (all-cause mortality and rates of surgical thrombectomy) that were reported by a sufficient number of studies. For the other outcomes (ICU length of stay, hospital length of stay) that were reported by a small number of studies, sensitivity analyses were not performed.

The publication bias for the outcomes of mortality was assessed via funnel plot and Begg’s and Egger’s tests. When the *p*-values for both Begg’s and Egger’s tests were >0.05, then there was a low likelihood of publication bias. The funnel plot assessed the association between study size (*Y*-axis as Standard Error) as a function of the effect size (*X*-axis). Smaller studies appeared toward the bottom of the funnel plot.

We used the I^2^ value and Cochrane’s Q-statistic to measure heterogeneity. The I^2^ value indicates the percentage of variance between the included studies’ effect sizes. The Q-statistic tests for the null hypothesis that the overall effect size of this meta-analysis would be similar to the true effect size from a hypothetical study involving millions of studies. Thus, both the I^2^ and the Q-statistic measure different levels of heterogeneity while complementing each other.

These small studies have more sampling variation and tend to have larger than average effects, which makes them more likely to have statistical significance. The studies are distributed symmetrically around the overall effect size, in the absence of publication bias, as studies reporting negative and positive results were published and included.

Since we anticipated the presence of heterogeneity from this meta-analysis, we also performed moderator analysis, using the categorical variables of each study’s demographic information (study design, study settings, sample size of total patients) to assess the source of heterogeneity and to compare subgroups. We hypothesized that in studies using retrospective methodology, enrolling patients in the ED would be associated with higher heterogeneity than in studies with prospective settings or patients who were admitted for PE treatment. We were also interested in whether any interventions received by PERT patients would have any association with mortality. Therefore, we performed exploratory univariable and multivariable metaregressions using the number of interventions among the PERT patients as continuous independent variables (percentages of patients undergoing anticoagulation, surgical thrombectomy, catheter-directed thrombolysis, number of anticoagulations, number of extracorporeal membrane oxygenation [ECMO]). We planned these analyses a priori because we hypothesized that patients with PE and who received advanced surgical therapy would be correlated with lower mortality, while patients undergoing ECMO would be associated with higher mortality as they most likely sustained massive PE and hemodynamic collapse. The results from this exploratory method regressions were reported as coefficient correlations, 95% Cis, and the associated *p* values.

All random-effect meta-analyses and sensitivity analyses were performed with the software Comprehensive Meta-Analysis Version 4 (www.meta-analysis.com, Englewood, NJ, USA). For all statistical analyses, a *p*-value < 0.05 was considered statistically significant.

## 3. Results

### 3.1. Study Description

**Figure 1:**
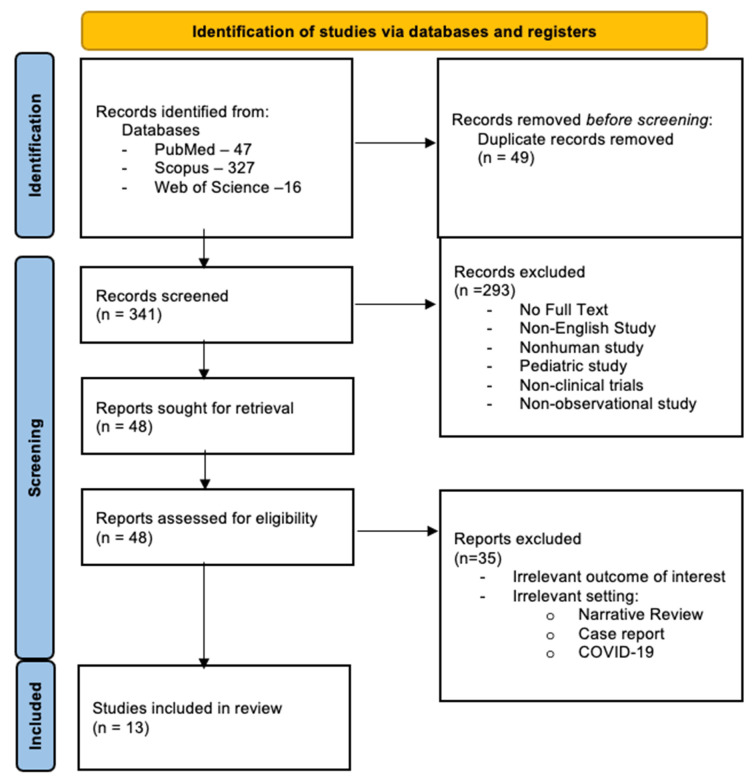
PRISMA 2020 flow diagram for new systematic reviews which included searches of databases and registers only. PRISMA study selection diagram [12].

The search for eligible studies yielded a total of 390 titles and abstracts (Figure 1). After full-text screening, we included 13 studies in our analysis. Two studies were prospective [15,16], while 11 studies were retrospective observational [17,18,19,20,21,22,23,24,25,26,27]. Most of the studies involved patients in the ED [15,18,19,20,23,24,25,27], while four studies involved both ED and inpatient settings [16,17,22,26].

The Newcastle–Ottawa Scale was used to assess the quality of all included studies, as all were observational studies (Table 1).

### 3.2. Summary of Studies

The included studies involved a total of 12,586 patients: 7512 (60%) patients were from the pre-PERT period and 5065 (40%) patients were from the PERT period. One study [15] did not report the number of female patients, but the reported percentages of total female patients ranged from 8% [25] to 53% [16]. While most studies compared the periods of pre-PERT and PERT, other studies [17,18,20,21,22,24] compared the outcomes of patients for whom PERT was activated, compared to those in the pre-PERT period or for whom PERT was not activated. The mean (+/−Standard Deviation [SD]) age for the pre-PERT group was 62 (+/−3) years, and the age for the PERT group was 61 (+/−3, *p* = 0.85) years. Most studies did not report the acuity of their patients such as the Pulmonary Embolism Severity Index (PESI) score or the BOVA.

### 3.3. All-Cause Mortality

Twelve studies reported the rate of all-cause mortality for their patient populations, except the study by Parikh et al. [15].

**Figure 2:**
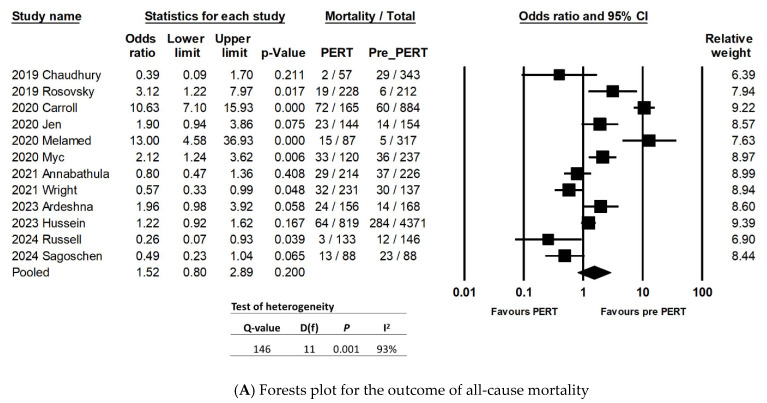

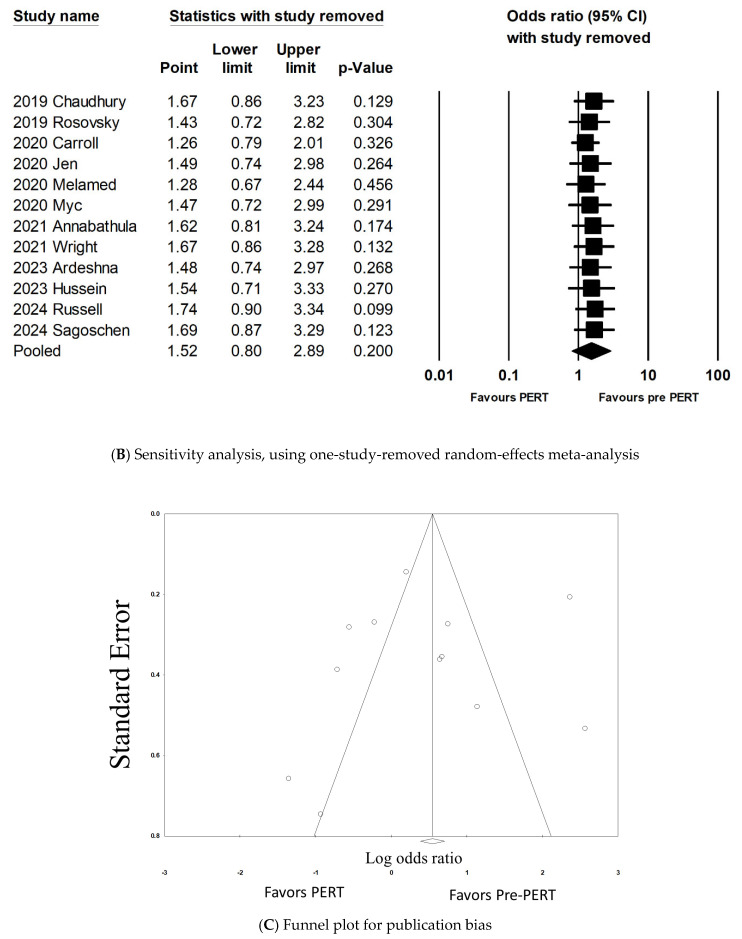
(**A**) Forests plot for the outcome of all-cause mortality [11,16,17,18,19,20,21,22,23,24,25,26]. (**B**) Sensitivity analysis, using one-study-removed random-effects meta-analysis for the outcome of all-cause mortality [11,16,17,18,19,20,21,22,23,24,25,26]. (**C**) Funnel plot for publication bias among studies being included for the outcome of all-cause mortality.

Patients in the PERT period were associated with similar odds of all-cause mortality as patients in the pre-PERT period (OR: 1.52; 95% CI: 0.80–2.89; *p* = 0.20). There was high heterogeneity between studies with both the Q-statistic (*p* = 0.001) and I^2^ value (94%) (Figure 2A). The sensitivity analysis with one-study-removed random-effects meta-analysis suggested that there were no individual studies that would affect the overall outcome of the population’s effect size (Figure 2B).

The funnel plot (Figure 2C) shows an equal distribution of the number of studies on both sides of the plot, which suggests that there was no publication bias among the studies in this meta-analysis. The result was also confirmed by both Begg’s and Egger’s tests, for which *p*-values were 0.94 and 0.89, respectively.

Moderator analyses, using three different variables for subgroups (study design, study setting, and study sample size) showed that PERT patients in retrospective studies were associated with higher odds of all-cause mortality, compared to those in the pre-PERT period. Similarly, patients in the ED settings and PERT period were associated with higher odds of mortality (OR: 2.56; 95% CI: 1.19–5.5), whereas PERT patients in studies involving mixed settings (ED/inpatient) were associated with lower odds of mortality (OR: 0.58; 95% CI: 0.37–0.89), and this difference was statistically significant (*p* = 0.001) (Appendix A). Studies with different sample sizes did not show any difference in the odds of mortality between pre-PERT and PERT patients. Only the subgroup of studies that involved both ED and inpatient patients was associated with low heterogeneity (I^2^ value= 12%), when compared to the subgroup of studies involving ED setting only (I^2^ = 94%). The studies with both ED and inpatient settings also reported that PERT patients were associated with lower odds of all-cause mortality (OR: 0.58; 95% CI: 0.37–0.89; *p* = 0.016), while the studies in the ED setting reported that PERT patients were associated with higher odds of mortality, when compared to pre-PERT patients (OR: 2.56; 95% CI: 1.19–5.5; *p* = 0.016) (Table 2).

Exploratory multivariate meta-regressions including the ages of PERT patients and the percentages of female in the PERT population demonstrated that higher percentages of females, among the PERT population, were positively correlated (correlation coefficient: 0.17; 95% CI: 0.004 to 0.34; *p* = 0.045) with higher odds of all-cause mortality (Appendix B). In contrast, in multivariate meta-regression using both the percentages of PERT patients undergoing surgical thrombectomy and catheter-directed thrombolysis, higher percentages of surgical thrombectomy were negatively correlated with all-cause mortality (correlation coefficient: −0.15; 95% CI: −0.3 to −0.001; *p* = 0.048) (Table 2).

### 3.4. ICU Length of Stay

**Figure 3:**
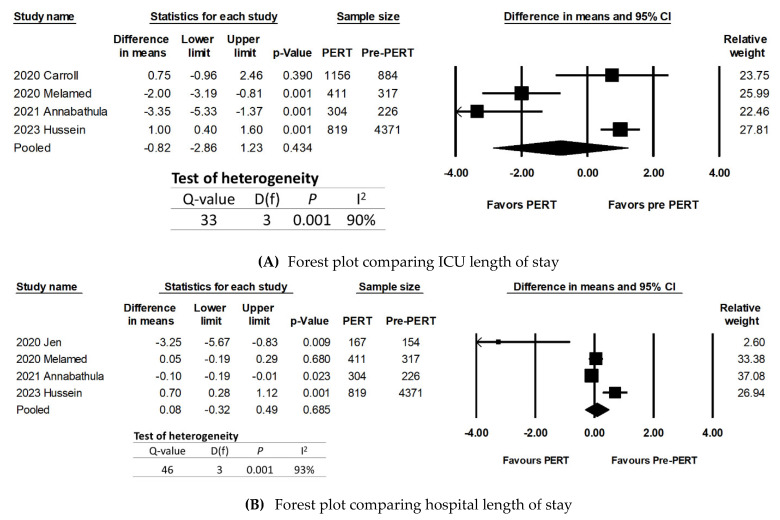
Results of random-effects meta-analysis, comparing the length of stay in the ICU and the hospital, in days, between PERT patients versus non-PERT patients. (**A**) Forest plot comparing ICU length of stay [18,20,22,25]. (**B**) Forest plot comparing hospital length of stay [19,20,22,25].

Four studies [19,20,22,25] reported the ICU length of stay for their patients. Two studies [19,22] reported that PERT patients had shorter ICU lengths of stay than pre-PERT patients, while Melamed et al. [20] and Hussein et al. [25] reported PERT patients were associated with a longer ICU length of stay. In the random-effects meta-analysis, there was no significant difference in ICU length of stay between PERT and pre-PERT patients (difference in means: 0.08; 95% CI: −0.32 to 0.49; *p* = 0.68). There was significant heterogeneity with both the Q-statistic (*p*-value < 0.001) and I^2^ value (I^2^ = 93%) (Figure 3).

Moderator analyses and meta-regression were not performed due to the small number of studies.

### 3.5. Hospital Length of Stay

Four studies [18,20,22,25] reported the hospital length of stay for their patients. Two studies [18,25] reported that PERT patients were associated with a longer hospital length of stay than pre-PERT patients. The random-effects meta-analysis demonstrated that there was no statistically significant difference in hospital length of stay between PERT versus pre-PERT patients (difference in means: −0.82; 95% CI: −2.86 to 1.23; *p* = 0.43) (Figure 3B). There was significant heterogeneity with both the Q-statistic (*p* < 0.001) and I^2^ value (90%). Both *p*-values for Begg’s test and Egger’s test were 0.73 and 0.27, respectively, which demonstrated that publication bias was less likely for this subgroup analysis.

Moderator analyses and meta-regression were not performed due to the small number of studies.

### 3.6. Rates of Surgical Thrombectomy

**Figure 4:**
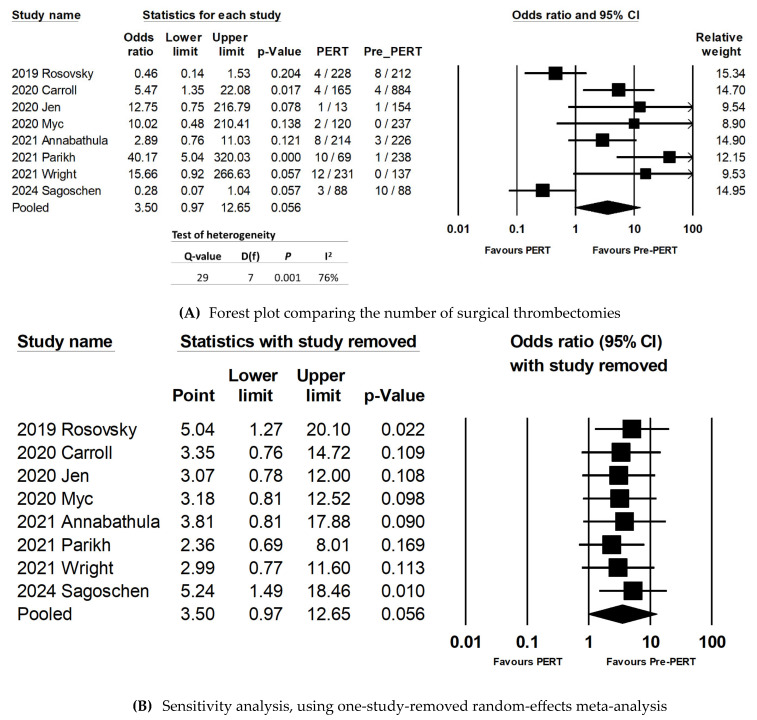
Results of random-effects meta-analysis, comparing the number of surgical thrombectomies between PERT patients versus non-PERT patients. (**A**) Forest plot comparing the number of surgical thrombectomies [11,15,16,18,19,21,22,23]. (**B**) Sensitivity analysis, using one-study-removed random-effects meta-analysis for the number of surgical thrombectomy [11,15,16,18,19,21,22,23].

Eight studies reported the rate of surgical thrombectomy [15,16,18,19,21,22,23,27]. The rates of surgical thrombectomy were not statistically significant among PERT versus pre-PERT patients (OR: 3.50; 95% CI: 0.97–12.65; *p* = 0.056) (Figure 4A). Sensitivity analysis with one study removed did not show any individual study affecting the overall effect size of the study (Figure 4B). Both *p*-values for Begg’s test and Egger’s test were 0.27 and 0.07, respectively, which suggested that publication bias was less likely for this subset of studies. The Begg’s test *p*-value was 1.0 and Egger’s test *p*-value was 0.30, which suggested that publication bias is less likely to be present for this subgroup meta-analysis.

Moderator analyses, using three different variables for subgroups (study design, study setting, and study sample size) showed that all subgroups were associated with high heterogeneity (Appendix B), and there was no statistical difference in the rates of surgical thrombectomy between PERT versus pre-PERT patients in different subgroups.

No meta-regression was performed for this outcome, due to the lack of common variables.

## 4. Discussion

In this random-effects meta-analysis of 13 observational studies, we demonstrated that there was no statistical difference among all-cause mortality, the rates of surgical thrombectomy, or the length of stay between PERT versus pre-PERT patients.

There was significant heterogeneity among the studies within this meta-analysis. The heterogeneity could be explained by the fact that the authors selected their patients differently; for example, certain studies would only include patients with massive and submassive PE, while other studies would include all patients with PE. Therefore, the rates of all-cause mortality or surgical thrombectomy would be reported differentially, as patients with massive PE would be associated with higher risk for mortality and needs of surgical thrombectomy. This study was able to identify a potential source of heterogeneity, which was the setting of the included studies. All four studies involving both ED and inpatient settings [16,17,22,26] demonstrated that PERT patients were associated with lower odds of mortality, compared to pre-PERT patients. In contrast, there was disagreement between the studies involving only ED patients regarding odds of mortality. Wright et al. [23] suggested that PERT patients were associated with lower odds of mortality, while all other studies disagreed [18,19,20,21,24,25,27]. This heterogeneity in this meta-analysis highlights the need for a consensus of what future studies should report to improve our understanding of the efficacy of PERTs.

The studies included in this analysis had limited reporting of measures of PE severity such as PESI or BOVA scores, biomarker levels, or the presence of imaging findings demonstrating right heart strain. One study that did report these, by Chaudhury et al., noted that the mortality benefit found was most prominent in high-risk patients [17]. Improving the reporting of risk stratifying measures in patients included in future studies would allow for future analyses to better investigate this potential effect.

There are a number of factors that the PERT system may affect that were not able to be examined in this review such as time to treatment, rate of hospital readmission, and longer-term measures of mortality. One of the studies included in this review by Myc et al. in 2020 did include readmission as a metric in their comparison and found that there was a statistically significant difference in a rate of 30-day readmissions [21].

### Limitations

This meta-analysis has several limitations. Although the analysis suggests there was low likelihood of publication bias, all of the included studies were observational, so there would be risk of selection bias. Due to the pre–post nature, most of the studies did not report detailed clinical information about their patients; thus, we were not able to perform more thorough analyses to adjust for more confounding factors. Certain studies also chose to have their PERT assess all PE patients, which may have resulted in low-risk PEs skewing the results; specifically, eight studies focused on intermediate- and high-risk PE, two studies included a low-risk population, and three others did not specify whether low-risk patients were included. Furthermore, although our subgroup of small studies with less than 350 patients did not report statistically significant different rates of mortality, they did suggest lower odds of mortality, compared to studies with a larger sample size. This may suggest that a small study effect, which suggests a benefit of PERTs, may be present, and we did not have a large number of studies to confirm it.

## 5. Conclusions

The use of pulmonary embolism response teams has been gaining more widespread use both in the US and internationally with the purpose of improving the process for treating patients with high-risk pulmonary emboli. Despite their popularity, our meta-analysis demonstrated no significant difference in all studied measures in the pre- and post-PERT time periods, which notably included patient mortality and length of stay. Despite there being no significant difference in mortality, the authors of this review believe there is a potential value to patients that warrants further investigation. The early assembly of a specialized team can expedite PE risk stratification steps like echocardiography and, as was noted by Wright et al. in 2019, decrease the time to advanced interventions and reduce ED length of stay [25]. As noted above, the level of heterogeneity of the studies when reporting outcomes and interventions also points to the potential for an institution-dependent result of the PERT process on patient outcomes. Further study into the details of the PERT system at institutions reporting mortality benefits may reveal practice differences that explain the outcome discrepancy and could help optimize PERT implementation at other institutions. Further investigation is warranted if the PERT process may have an effect on other metrics such as time intervals to intervention or hospital readmission. Additional studies using randomized, controlled trial designs may also provide more clarity of if a PERT has a measurable benefit.

## Figures and Tables

**Table 1:** Demographics of studies included.

Study (Year, Author)	Total NOS Score	Setting	Number of Patients	No of PERT Activations	Study Type	Mean Age (Pre-PERT/Post-PERT)	% Female (Pre-PERT/Post-PERT)	Mortality (Pre-PERT/Post-PERT)	Thrombolytic (Pre-PERT/Post-PERT)	IVC Filter (Pre-PERT/Post-PERT)	Surgical/Mechanical Thrombectomy (Pre-PERT/Post-PERT)	CDT (Pre-PERT/Post-PERT)	Any Anticoagulation (Pre-PERT/Post-PERT)	DOAC (Pre-PERT/Post-PERT)	ECMO (Pre-PERT/Post-PERT)
2019, Chaudhury et al. [17]	8	ED/inpatient	769	57	Retrospective Cohort Study	58.1	49.3	29	4	76	4	1	318	50	0
			Pre PERT: 343			57.2	47.9	20	14	70	3	6	410	50	2
			Post PERT: 426												
2019, Rosovsky et al. [11]	6	ED	440	228	Retrospective Cohort Study	59	52	6	10		9	10			
			Pre PERT: 212			61	47	19	12		4	31			
			Post PERT: 228												
2020, Carroll et al. [18]	8	ED	2042	165	Retrospective Cohort Study	62.3	52.3	60	34	95	4	10	836	6	4
			Pre PERT: 884			63.6	53.9	72	24	80	4	35	1088	30	5
			Post PERT: 1158												
2020, Jen et al. [19]	8	ED	321	167	Retrospective Cohort Study	61.1	51.9	14	2		1			9	
			Pre PERT: 154			60.3	51.5	23	6		1	4		25	1
			Post PERT: 167												
2020, Melamed et al. [20]	8	ED	728	87	Retrospective Cohort Study	62.4	52.4	5	8			9			
			Pre PERT: 317			62.4	47.7	15	90			12			
			Post PERT: 411												
2020, Myc et al. [21]	7	ED	554	120	Retrospective Cohort Study	62	48	36	0	53	0	7		113	0
			Pre PERT: 237			63.5	48.3	33	5	41	2	26		200	4
			Post PERT: 317												
2021, Annabathula et al. [22]	8	ED/inpatient	530	214	Retrospective Cohort Study	59.5	58.4	37	10		3	46	221	23	12
			Pre PERT: 226			58.1	53	29	7		8	55	298	145	12
			Post PERT:												
2021, Parikh et al. [15]	9	ED	307	69	Prospective Cohort Study	60.1									
2021, Wright et al. [23]	8	ED	368	231	Retrospective Cohort Study	63.2	48	33	11	9	0	1	134		1
			Pre PERT: 137			63.9	46	46	23	14	12	10	227		2
			Post PERT: 231												
2023, Ardeshna et al. [24]	8	ED	644	156	Retrospective Cohort Study	58	53	14.28	2	9		14	156	5	
			Pre PERT: 168			59	51	24	8	15		10	447.44	9.52	
			Post PERT: 476												
2023, Hussein et al. [25]	8	ED	5190	819	Retrospective Cohort Study	62.6	52.6								
			Pre PERT: 4371			59.8	47								
			Post PERT: 819												
2024 Russell et al. [26]	7	ED/inpatient	279	133	Retrospective Cohort Study	63	48	12	7						
			Pre PERT: 146			61	41	3	6		21	48	132		
			Post PERT: 133												
2024 Sagoschen et al. [16]	8	ED/inpatient	176	88	Prospective Cohort Study	67	50	23	12		10				
			Pre PERT: 88			68	49	13	8		3				
			Post PERT: 88												

**Table 2:** Meta-regression using continuous variables for the primary outcome of all-cause mortality.

Variables	Number of Studies	Corr. Coeff. (95% CI)	*p*	R^2^	I^2^
PERT age	12	0.04 (−0.15 to 0.23)	0.72	0.2	89%
PERT number of females		0.17 (0.004 to 0.34)	0.045		
PERT thrombolysis	10	0.03 (−0.0002 to 0.06)	0.051	0.19	92%
PERT IVC filter	5	0.04 (0.02 to 0.057)	0.001	0.88	65%
PERT surgical thrombectomy	7	0.01 (−0.04 to 0.07)	0.66	0.23	93%
PERT catheter-directed thrombolysis		−0.15 (−0.3 to −0.001)	0.048		
PERT number of anticoagulations	6	0.003 (0.0027 to 0.0039)	0.001	1	0%
PERT number of ECMO	5	−0.04 (−0.37 to 0.3)	0.84	0	96%

## Data Availability

There is no proprietary data for this research. All data collected from the referenced articles was published and available to the public.

## Appendix A. Search Strategy

PubMed (number of results = 47)

(“Pulmonary Embolism Response Team” OR PERT) AND (“Pulmonary Embolism”[Mesh] OR “Anticoagulants”[Mesh] OR “Tissue Plasminogen Activator/therapeutic use”[Mesh] OR “Thrombolytic Therapy”[Mesh] OR “Fibrinolytic Agents”[Mesh] OR “Fibrinolytic Agents”[Pharmacological Action] OR “Thrombectomy”[Mesh] OR “Embolectomy”[Mesh] OR “Catheterization, Swan-Ganz”[Mesh] OR “Emergency Service, Hospital”[Mesh] OR “Emergency Treatment”[Mesh] OR “Intensive Care Units”[Mesh] OR “Hospital Rapid Response Team”[Mesh] OR “Patient Care Team”[Mesh] OR “Time-to-Treatment”[Mesh] OR “Time Factors”[Mesh] OR “Workflow”[Mesh]) AND (“Adult”[Mesh] OR “Middle Aged”[Mesh] OR “Aged”[Mesh]) NOT (“Case Reports”[Publication Type] OR “Technical Report”[Publication Type] OR “case series” OR “adult series” OR perturbation OR refractory OR perinatal OR ticagrelor OR pancreatic OR estrogen OR endocrine OR asthma OR “Thiadiazoles”[Mesh])

Cochrane CENTRAL (0 trials)

(“Pulmonary Embolism Response Team” OR PERT ) AND ([mh “Pulmonary Embolism”] OR [mh Anticoagulants] OR [mh “Tissue Plasminogen Activator/therapeutic use”] OR [mh “Thrombolytic Therapy”] OR [mh “Fibrinolytic Agents”] OR “Fibrinolytic Agents” OR [mh Thrombectomy] OR [mh Embolectomy] OR [mh “Catheterization, Swan-Ganz”] OR [mh “Emergency Service, Hospital”] OR [mh “Emergency Treatment”] OR [mh “Intensive Care Units”] OR [mh “Hospital Rapid Response Team”] OR [mh “Patient Care Team”] OR [mh Time-to-Treatment] OR [mh “Time Factors”] OR [mh Workflow]) AND ([mh Adult] OR [mh “Middle Aged”] OR [mh Aged]) NOT (“Case Reports”:pt OR “Technical Report”:pt OR “case series” OR “adult series” OR perturbation OR refractory OR perinatal OR ticagrelor OR pancreatic OR estrogen OR endocrine OR asthma OR [mh Thiadiazoles])

Scopus Advanced Search (number of results = 327)

ALL(“Pulmonary Embolism Response Team”) AND (INDEXTERMS(“Pulmonary Embolism”) OR INDEXTERMS(Anticoagulants) OR INDEXTERMS(“Tissue Plasminogen Activator”) OR INDEXTERMS(“Thrombolytic Therapy”) OR INDEXTERMS(“Fibrinolytic Agents”) OR ALL(“Fibrinolytic Agents”) OR INDEXTERMS(“Thrombectomy”) OR INDEXTERMS(“Embolectomy”) OR INDEXTERMS(“Catheterization”) OR INDEXTERMS(“Emergency Service”) OR INDEXTERMS(“Emergency Treatment”) OR INDEXTERMS(“Intensive Care Units”) OR INDEXTERMS(“Hospital Rapid Response Team”) OR INDEXTERMS(“Patient Care Team”) OR INDEXTERMS(“Time-to-Treatment”) OR INDEXTERMS(“Time Factors”) OR INDEXTERMS(Workflow)) AND (INDEXTERMS(Adult) OR INDEXTERMS(“Middle Aged”) OR INDEXTERMS(Aged)) AND NOT TITLE(“Case Report” OR “Technical Report” OR “case series” OR “adult series”)

Web of Science Advanced Search (number of results = 16)

ALL=(“Pulmonary Embolism Response Team” OR “PERT”) AND ALL=(“Pulmonary Embolism” OR “Anticoagulant” OR “Tissue Plasminogen Activator” OR “Thrombolytic Therapy” OR “Fibrinolytic Agent” OR “Thrombectomy” OR “Embolectomy” OR “Catheterization” OR “Emergency Department” OR “Emergency Treatment” OR “Intensive Care Unit” OR “Hospital Rapid Response Team” OR “Patient Care Team” OR “Time-to-Treatment” OR “Time Factors” OR “Workflow”) AND ALL=(“Adult” OR “Middle Aged” OR “Aged”) NOT TI=(“Case Report” OR “Technical Report” OR “case series” OR “adult series”).

## Appendix B. Moderator Analyses Subgroups Using Categorical Variables and the Outcome of All-Cause Mortality

**Table:**

Variables		Meta-Analysis					Heterogeneity	
		Number of Studies	Outcome	95% CI	*p*	Q-Value	D(f)	*p*
Outcome: all-cause mortality								
Study design	Prospective	1	0.49	0.23–1.04	0.065	NA	NA	NA
	Retrospective	11	1.69	0.8–3.29	0.12	135	10	0.001
Study settings	ED	8	2.56	1.19–5.5	0.016	110	7	0.001
	ED/Inpatient	4	0.58	0.37–0.89	0.014	3	3	0.33
Study sample size	<350 patients	3	0.67	0.2–2.13	0.5	10	2	0.006
	351–700 patients	5	1.36	0.74–2.53	0.33	19	4	0.001
	>701 patients	4	3.02	0.66–13.8	0.15	89	3	0.001
Outcome: surgical thrombectomy								
Study design	Prospective	2	3.11	0.02–410	0.65	16	1	0.001
	Retrospective	6	3.51	1.06–11.6	0.04	12	5	0.029
Study settings	ED	6	6.38	1.28–31.6	0.023	19	5	0.002
	ED/Inpatient	2	0.89	0.089–8.90	0.921	6	1	0.015
Study sample size	<350 patients	3	4.7	0.14–154	0.39	18	2	0.001
	351–700 patients	4	2.6	0.52–12.9	0.24	9	3	0.033
	>701 patients	1	3.46	1.35–22.1	0.017	NA	NA	NA
